# The collaborations among healthcare systems, research institutions, and industry on artificial intelligence research and development

**DOI:** 10.3389/frai.2025.1694145

**Published:** 2026-01-12

**Authors:** Jiancheng Ye, Michelle Ma, Malak Abuhashish

**Affiliations:** 1 Weill Cornell Medicine, Cornell University, New York, NY, United States; 2 Macaulay Honors College in Hunter College, New York, NY, United States

**Keywords:** artificial intelligence, collaboration, data privacy, healthcare system, implementation research, industry standards, research and development

## Abstract

**Objectives:**

The integration of Artificial Intelligence (AI) in healthcare promises to revolutionize patient care, diagnostics, and treatment protocols. Collaborative efforts among healthcare systems, research institutions, and industry are pivotal to leveraging AI’s full potential. Understanding these dynamics is essential for addressing current challenges and shaping future AI development in healthcare. This study aims to characterize collaborative networks and stakeholders in AI healthcare initiatives, identify challenges and opportunities within these collaborations, and elucidate priorities for future AI research and development.

**Methods:**

This study analyzed publicly available survey data previously collected by the Chinese Society of Radiology and the Chinese Medical Imaging AI Innovation Alliance. We performed secondary analysis of the national cross-sectional survey that was conducted in China with a total of 5,262 participants (5,142 clinicians and 120 research institution professionals), involving participants from three key groups: clinicians, institution professionals, and industry representatives. The survey explored diverse aspects including current AI usage in healthcare, collaboration dynamics, challenges encountered, and research and development priorities.

**Results:**

Findings reveal high interest in AI among clinicians, with a significant gap between interest and actual engagement in development activities. Key findings include limited establishment of AI research departments and scarce interdisciplinary collaborations. Despite the willingness to share data, progress is hindered by concerns about data privacy and security, and lack of clear industry standards and legal guidelines. Future development interests focus on lesion screening, disease diagnosis, and enhancing clinical workflows.

**Conclusion:**

This study highlights an enthusiastic yet cautious approach toward AI in healthcare, characterized by significant barriers that impede effective collaboration and implementation. Recommendations emphasize the need for AI-specific education and training, secure data-sharing frameworks, establishment of clear industry standards, and formation of dedicated AI research departments.

## Introduction

The advent of Artificial Intelligence (AI) in healthcare represents a paradigm shift, promising unprecedented advancements in medical diagnostics, patient care, and treatment methodologies (Jiang et al., 2017). AI integration into healthcare is a multifaceted endeavor extending beyond sophisticated algorithm development or cutting-edge technology deployment (Allioui and Mourdi, 2023). It encompasses a broad spectrum of activities including diagnostic imaging enhancements, personalized medicine tailoring, predictive analytics for patient outcomes, and automation of clinical decision-making processes (Zhang et al., 2025; Zhang S. et al., 2025). These applications are transforming the conceptual framework of healthcare delivery while setting new benchmarks for efficiency, accuracy, and patient-centric care (Wang et al., 2025). AI technology integration into healthcare is not merely a technological leap; it is an intricate process requiring harmonious collaborative efforts among various stakeholders (Albahri et al., 2023).

Harnessing AI’s full potential in healthcare requires strategic collaboration among key stakeholders: healthcare practitioners who contribute clinical expertise and insights, research institutions that drive innovation through rigorous scientific inquiry, and industry stakeholders that translate technological advancements into viable healthcare solutions (Bajwa et al., 2021). This tripartite collaboration is pivotal for overcoming translational hurdles that often hinder seamless AI technology integration into clinical settings, ensuring that AI innovations are not only technologically robust but also aligned with practical healthcare needs, making them readily adoptable in real-world scenarios (Ye, 2021a).

However, fostering effective collaboration among these diverse entities presents significant challenges. Disparate objectives, varying operational cultures, and rapid AI technology evolution often lead to misalignments that can impede collaborative efforts (Lin-Greenberg, 2020). Additionally, regulatory considerations, ethical concerns, and data privacy issues add complexity layers to these partnerships (Chen et al., 2024). Navigating these challenges requires deep understanding of the collaborative ecosystem, clear articulation of common goals, and establishment of frameworks that facilitate mutual engagement and benefit sharing.

To unravel collaboration intricacies in the AI healthcare domain, this study relies on data from the Chinese Society of Radiology and the Chinese Medical Imaging AI Innovation Alliance (Chinese Society of Radiology, n.d.). These organizations serve as repositories of valuable insights, encapsulating experiences and perspectives of clinicians, researchers, and industry professionals. Through a meticulously designed national cross-sectional survey, we aim to illuminate collaborative relationships that define the current state of AI in healthcare. This paper seeks to achieve several key objectives: (1) explore existing networks and partnerships among clinicians, research institutions, and industry stakeholders engaged in AI healthcare initiatives; (2) uncover challenges faced by stakeholders in AI adoption and implementation in healthcare while identifying opportunities for overcoming these hurdles; (3) provide insights into priorities and areas of interest for future research and development in the AI healthcare domain.

This study examines the intricate dynamics of collaboration among healthcare systems, research institutions, and industry stakeholders, providing a comprehensive overview of the current landscape, inherent challenges, and prospective directions in AI research and implementation within healthcare.

## Methods

Data utilized in this study were obtained from publicly available datasets released by two prominent Chinese organizations: the Chinese Society of Radiology and the Chinese Medical Imaging AI Innovation Alliance (Chinese Society of Radiology, n.d.). The original survey was distributed electronically through the societies’ networks and professional contacts across China (Xiao and Liu, 2019). We extracted and analyzed the de-identified aggregate data from the publicly released dataset. These datasets compile comprehensive information related to AI research and implementation in healthcare, with specific emphasis on radiology and medical imaging. Survey participants were meticulously selected to ensure diverse perspectives from key stakeholders in the AI healthcare ecosystem. The study included individuals from three distinct groups: clinicians, researchers from renowned healthcare research institutions, and representatives from the healthcare industry. This stratification was essential to capture a holistic view of the collaborative landscape among healthcare systems, research institutions, and industry players. Descriptive statistics were performed to derive meaningful insights from survey responses.

The clinician group comprised healthcare professionals actively engaged in clinical practice, including radiologists, physicians, and other specialists. Their firsthand patient care experience provided valuable insights into practical applications and challenges of AI technologies in clinical settings. Research institutions group participants were affiliated with prominent healthcare research institutions across China. These individuals brought extensive knowledge and expertise in innovative research and development within the AI healthcare domain, contributing to academic and scientific aspects of the study. The industry group consisted of professionals from companies and organizations actively involved in development, deployment, and commercialization of AI solutions in healthcare. This group’s perspective illuminated technological advancements, market trends, and industry needs in the AI healthcare sector.

To gather comprehensive and relevant information, a structured survey instrument was designed collaboratively by experts in healthcare, AI, and survey methodology. The survey questionnaire encompassed a range of topics including: (1) current AI usage in healthcare—examining current integration of AI technologies in healthcare practices; (2) challenges and barriers—identifying challenges faced by clinicians, researchers, and industry professionals in AI adoption and implementation in healthcare; (3) collaboration dynamics—investigating existing collaborations, partnerships, and communication channels among healthcare systems, research institutions, and industry stakeholders in the AI domain; and (4) research and development priorities—understanding key areas of interest and priorities for future research and development in AI healthcare. The survey included structured questions asking clinicians to indicate their preferences for various types of support, rank their priorities for AI development focus areas, and identify the most significant barriers they encountered. While the survey primarily employed closed-ended questions with predetermined response categories, the comprehensive scope of topics covered allowed for synthesis of actionable recommendations based on the patterns and priorities emerging from clinicians’ responses across multiple question domains. Questions allowed single-choice responses (for mutually exclusive options such as demographic information), multiple-choice responses (for opinions and interests where participants could select all applicable options), or Likert scale ratings. Tables presenting results from multiple-choice questions may show totals exceeding 100%.

### Study approval

This study utilized exclusively publicly available, de-identified aggregate data released by the Chinese Society of Radiology and the Chinese Medical Imaging AI Innovation Alliance.

## Results

### Clinicians group

#### Characteristics of participants

In our study, we distributed 5,148 questionnaires within the clinician group, achieving a 99.9% response rate (5,142 responses). These surveys spanned 31 regions nationwide, incorporating 2,135 hospitals. The most represented age group among clinicians was 30–40 years (34%), closely followed by 40–50 years (33%). This age distribution was consistent across both secondary and tertiary hospitals.

Respondents’ educational qualifications provide valuable insights into their academic backgrounds. The predominant qualification was a bachelor’s degree (58%), followed by master’s degrees (22%) and professional or doctorate degrees (20%). Notably, half of the clinicians, especially those with keen interest in AI, were attending physicians or deputy chief physicians with over 15 years of radiology experience. Among these, 66% worked in secondary hospitals and 50% in tertiary hospitals.

Professional roles varied, with 27% of participants serving as department directors and 13% as deputy department heads. Primary clinical research areas were diverse, focusing on abdomen (56%), chest (45%), bone and joints (36%), nervous system (35%), and head and neck (29%), with additional interests in breast, pediatric, interventional radiology, and molecular imaging.

#### Current state of health information systems and AI collaboration

Table 1 presents the current state of health information systems and AI collaboration in the clinician group. Overall, 47% of hospitals lack structured imaging report systems, 43% are planning implementation, and only 10% have established and actively use such systems. These systems are predominantly applied in departments addressing lung nodules or lung cancer, colorectal cancer, breast cancer, and coronary artery disease. Regarding hospital informatization, 63% of patient information is accessible through unified systems, 31% requires querying across multiple systems, and 6% remains inaccessible.

**Table 1:** Current state of AI collaboration in healthcare systems from the clinician group.

Characteristics	Percentage
Structured imaging report system	
Not established	47%
Planning to establish	43%
Already established and operational	10%
Hospital informatization level	
Accessible within unified system	63%
Requires queries in different system	31%
Not accessible	6%
Involvement in AI-related research	
Not involved	74%
Participated but no results	21%
Published research papers	4%
Developed AI products	0.8%
Obtained related patents	0.5%
Research received domestic or international awards	0.4%
Clinician collaboration with research institutions	
No collaboration with enterprises or research institutions	84%
No collaboration with imaging device companies	92%

In terms of AI involvement, 74% of clinicians have not engaged in AI-related research. Among the remainder, 21% participated without producing results, 4% published research papers, 0.8% developed AI products, 0.5% secured related patents, and only 0.4% received domestic or international recognition. Additionally, 84% of clinicians have not collaborated with relevant enterprises or research institutions, and 92% have not worked with imaging device companies.

#### Infrastructure for AI research

The majority of hospitals (72%) lack departments dedicated to AI research. A significant portion of clinicians (27%) are uncertain about such departments’ existence within their institutions, with only 1% of hospitals having established specialized AI research departments. Within these facilities, 55% do not have departments focused on translating research findings into practical applications. Only 20% have established such departments, while 25% of clinicians are unsure of their presence. Tertiary hospitals exhibit comparatively more robust AI research infrastructure, with 27% hosting relevant departments, contrasting sharply with 8% in secondary hospitals. A concerning 79% of hospitals lack engineering personnel engaged in AI research. Of the limited departments that exist, 11% have 1–2 individuals with at least master’s degrees, and only 4% have staff of five or more with comparable qualifications.

#### Data sharing for AI research

Table 2 shows that clinicians primarily contribute to AI research collaborations through image data sharing (89%), clinical information provision (76%), assistance with image data labeling (70%), identification of clinical needs and issues (70%), and feedback on AI products (52%). The preferred AI collaboration method involves complimentary data sharing for joint research publications or patents, favored by 55% of respondents, followed by free data sharing in exchange for AI product discounts (25%), and intra-hospital sharing or paid purchases (20%). Regarding data privacy and security, a majority (74%) recognize the need for data anonymization and confidentiality agreements, while 23% are unsure of relevant policies, and 3% find these measures unnecessary.

**Table 2:** Data sharing and collaboration mechanisms in the clinician group.

Characteristics	Percentage
Resources for AI collaboration from clinicians	
Image data sharing	89%
Clinical Information	76%
Image data labeling assistance	70%
Clinical needs and issues	70%
AI Product feedback	52%
AI collaboration mechanisms	
Free data sharing for research papers or patent	55%
Free data sharing with AI product discounts	25%
Sharing within hospital or paid purchasing	20%
Opinions on desensitizing data and signing confidentiality agreements	
Necessary	74%
Unclear about relevant policies	23%
Unnecessary	3%
Clinicians’ engagement with AI-Related Products	
Aware of AI-related products but haven not utilized them	74%
Have used AI-related products	20%
Actively involved in product development	5%
Participated in product development with tangible results	1%

Despite widespread awareness, 74% of clinicians have only heard of AI-related products without actual usage. In contrast, 20% have utilized such products, 5% are engaged in AI product development, and only 1% have contributed to product development with concrete outcomes. AI products find their most significant application in lung nodule screening, utilized by 88% of hospitals, with coronary artery analysis (6%) and other areas like bone age, breast, and prostate diagnostics trailing in usage.

#### Challenges in the AI collaboration

Table 3 highlights challenges and barriers encountered in current AI collaborations. A significant 65% of clinicians identify absence of industry standards as a principal obstacle in AI research, while 63% cite lack of legal guidelines for employing AI products in clinical tasks. Moreover, 59% of clinicians report gaps in relevant AI knowledge. Concerns about AI product credibility and extensive workforce requirements are noted by 56 and 45% of respondents, respectively. Notably, 56% of clinicians express concerns that AI could lead to misdiagnoses or missed diagnoses, potentially resulting in critical medical errors. While 27% fear AI underperformance or failure, only 13% foresee no negative impacts on healthcare systems.

**Table 3:** Challenges in the AI collaboration in the clinician group.

Characteristics	Percentage
Perceived problems in AI research	
Lack of industry standards	65%
Lack of legal guidelines	63%
Lack of relevant knowledge in the AI field	59%
Low credibility of AI products	56%
Significant workforce requirements	45%
Beliefs about AI’s impact on healthcare systems	
May lead to misdiagnosis or missed diagnosis	56%
May perform poorly or fail to work	27%
Will have no negative impact on healthcare systems	13%

#### Future development in clinicians group

Table 4 presents prospects for future development in healthcare AI as perceived by the clinician group. A vast majority (90%) of clinicians anticipate needing substantial exploration time. Meanwhile, 25% are skeptical about achieving short-term practical results, 8% predict eventual replacement of radiologists, and 3% dismiss AI as a passing fad without practical utility. The primary interest area for healthcare AI among clinicians is lesion screening and detection (84%), followed by disease diagnosis (65%), and prognosis analysis and treatment effectiveness evaluation (64%). Medical education emerges as another significant interest area (41%). Clinicians also show enthusiasm for collaboration beyond research institutions, especially with industry (50% support) and forming internal AI teams (25%).

**Table 4:** Future development in the clinician group.

Characteristics	Percentage
Opinions on prospects of AI research	
Requires considerable time for exploration	90%
Practical results will not be achieved in the short term	25%
Radiologists will eventually be replaced	8%
Considered a passing trend with no practical value	3%
Interests in healthcare AI fields	
Lesion screening and detection	84%
Disease diagnosis	65%
Disease prognosis analysis and treatment effectiveness evaluation	64%
Medical education	41%
Expectations for collaboration	
Collaboration with research institutions	88%
Collaboration with enterprises	50%
Forming AI teams	25%
Desired resources for collaboration	
AI equipment and software	88%
Construction of image processing algorithms	73%
Research and funding support	60%
Training and education opportunities	0.3%
Expected time frame for research output	
Within 1 year	7%
Within 1–2 years	43%
Over 2 years	50%

Clinicians express strong preference for research institutions or technology companies to supply AI devices and software for clinical trials (88%), develop image processing algorithms (73%), and provide research and funding support (60%). A small fraction (0.3%) seeks additional resources like training and education opportunities. Most clinicians (93%) anticipate research output timelines exceeding 1 year, with expectations divided between 1 and 2 years (43%) and over 2 years (50%). Only 7% expect results within 1 year.

#### Recommendations from clinician group

Clinicians prioritize support through various avenues, with 82% identifying need for collaborative platforms with AI companies, 64% emphasizing the value of expert research teams, and 52% highlighting the importance of regular training workshops to disseminate AI knowledge. Drawing from patterns in clinicians’ reported challenges, expressed priorities, and stated preferences, five overarching recommendations emerge for advancing healthcare AI development. First, elevating training and knowledge levels is critical, with 59% of clinicians reporting gaps in relevant AI knowledge, indicating that expertise must reach grassroots hospitals for direct clinical application. Second, establishing platforms for multi-center cooperation would address the collaboration deficit, as 84% of clinicians currently have no partnerships with enterprises or research institutions, suggesting need for efficient resource sharing and communication among healthcare providers. Third, enhancing accuracy and usability of AI products directly responds to the 56% of clinicians expressing concerns about potential misdiagnoses or missed diagnoses, indicating efforts must strive to significantly reduce these risks. Fourth, creating comprehensive industry standards, legal frameworks, and dispute resolution mechanisms addresses the most frequently cited barrier, with 65% identifying absence of industry standards and 63% citing lack of legal guidelines, ensuring uniform AI approaches in healthcare. Fifth, implementing standardized data protection management practices would support the 74% of clinicians who recognize the necessity of data anonymization and confidentiality agreements, safeguarding patient information against unauthorized access.

### Research institutions group

A total of 120 surveys from research institutions across 19 regions nationwide were collected and analyzed. Age distribution shows a youthful skew: 69% under 30 years, 18% between 30 and 40 years, 10% between 40 and 50 years, and 3% over 50 years. Educationally, the group is highly qualified, with postgraduates comprising 58% and doctoral candidates, undergraduates, and college graduates making up the remainder. Notably, postgraduates and doctoral candidates together represent 91% of respondents. Healthcare AI research team sizes vary: 30% have 1–2 members, 28% have teams larger than 10, 23% work in groups of 6–10, and 19% operate in teams of 3–5. Leadership positions include 13% as research group heads, 6% as academic or institutional lab directors, and 2% leading provincial or ministerial key labs.

#### Current research status

Table 5 illustrates research status in the research institutions group. Predominant research areas are image classification/segmentation/target detection (42%), video image analysis (40%), and molecular imaging (36%). Other areas such as imaging methods, image reconstruction algorithms, reinforcement learning, and biometric identification each attract 12% focus. Less common fields include control systems and engineering, natural language processing, and autonomous driving, each with 5% share. Application-wise, lung nodule screening (32%), pathological diagnosis (25%), and early tumor diagnosis (25%) are most frequent, followed by breast disease screening (20%), stroke diagnosis (17%), and coronary heart disease diagnosis (12%). Emerging areas include retinal lesion screening, fracture screening, and bone age detection.

**Table 5:** Research status in the research institutions group.

Characteristics	Percentage
Research directions	
Image classification/segmentation/target detection	42%
Video image analysis	40%
Molecular imaging	36%
Imaging methods	12%
Imaging reconstruction algorithm	12%
Reinforcement learning	12%
Biometric identification	12%
Control systems and control engineering	5%
Natural language processing	5%
Autonomous driving	5%
Common application areas	
Lung nodule screening	32%
Pathological diagnosis	25%
Early tumors Diagnosis	25%
Breast disease screening	20%
Stroke diagnosis	17%
Coronary heart disease auxiliary diagnosis	12%
Primary issues addressed using AI technology	
Auxiliary diagnosis and clinical treatment decision-making	66%
Streamlining workflows and optimizing AI imaging methods	48%
Improving data security	18%
Achievements in AI-related research	
Journal papers	47%
Conference papers	37%
Obtained AI product patents	26%
Received domestic or international awards	70%
Involved in the AI research	30%

Primary research focus is assisting diagnosis and clinical treatment decisions (66%), with significant emphasis on enhancing clinical workflows and optimizing AI imaging methods (48%). Additionally, data security remains a concern for 18% of researchers. Regarding achievements, the majority resulted in scientific publications, with journal papers (47%) and conference papers (37%) leading. Additionally, 26% of researchers secured AI product patents, and 7% received domestic or international awards. Despite this, over 70% of researchers have not contributed to healthcare AI product development, with only 30% engaging in such projects.

#### Collaborations with healthcare systems

Table 6 provides an overview of collaborative landscape between research institutions and healthcare systems. One-third (33%) of researchers collaborated with just one hospital, while 31% have partnerships with 2–5 hospitals. Notably, another 31% have not engaged in prior collaborations. Collaborations extending to 6–10 hospitals or beyond are relatively rare, cumulatively accounting for only 5%. An important hurdle lies in obtaining meaningful data, as over one-third (33%) of researchers report not acquiring valuable data from collaborations. When data is accessible, it predominantly encompasses sample sizes from tens to hundreds of patients (22 and 26%, respectively). Only a minority access larger datasets, with 13% obtaining data from thousands of patients and 6% accessing data from over ten thousand patients.

**Table 6:** Collaborations between research institutions and healthcare systems from the research institutions group.

Characteristics	Percentage
Number of collaborative hospitals	
No prior collaboration	31%
Collaborate with 1 hospital	33%
Collaborate with 2–5 hospitals	31%
Collaborate with >5 hospitals	5%
Access to impactful data	
No impactful data	33%
Tens to hundreds of cases	22%
Hundreds to thousands of cases	26%
Several thousand cases	13%
Over ten thousand cases	6%
Collaboration with industry	
Do not collaborate with any healthcare systems	50%
Collaborate with <5 healthcare systems	39%
Collaborate with 5–10 healthcare systems	12%
Collaborate with >10 healthcare systems	2%
Collaboration with relevant companies	
Do not have collaborations with any relevant companies	47%
Collaborate with ≤5 companies	43%
Collaborate with >5 companies	10%

Half of researchers (50%) currently do not collaborate with any healthcare systems. Among those who do, the vast majority (39%) work with fewer than five systems. A smaller fraction engages with 5–10 systems (12%), and only 2% have partnerships with over 10 healthcare systems. Collaboration with relevant companies is also limited; nearly half report no such collaborations. Among those who collaborate, 43% work with fewer than five companies. Collaborations with 5–10 companies and more than 10 companies represent smaller portions, totaling 10%.

#### Data sharing and data security

Table 7 demonstrates data sharing and security in the research institutions group. Researchers exhibit clear preferences for data sharing mechanisms. A majority (55%) prefer sharing data without cost, resulting in co-authored research papers or patents, followed by 25% who prefer free data sharing coupled with AI product discounts, and 20% who engage in data sharing within hospital networks or paid purchases. Regarding data privacy and security, 74% of researchers affirm the necessity of data anonymization and confidentiality agreements. However, 23% remain uncertain about relevant policies, and 3% view these precautions as unnecessary.

**Table 7:** Data sharing and data security in the research institutions group.

Characteristics	Percentage
Data sharing methods in AI collaborations	
Free data exchange with sharing of research papers	72%
Free data sharing with discounted access to AI products	19%
Paid acquisition	9%
Concerns about data security	
Express importance of data security but lack certainty on how to ensure it	35%
Consider it crucial to understand and maintain processes for data security	30%
Show no particular concern about data security issues	20%
Possess key technologies for maintaining data security along with practical experience	15%

Among researchers, free data sharing for co-authored outputs emerges as most popular, capturing 72% approval, followed by 19% who prefer free data sharing with AI product discounts, and 9% who favor paid data acquisition. Despite recognizing data security importance, 30% of researchers admit uncertainty about achieving it effectively. Another 30% underscore the critical need for understanding and implementing robust data security measures, emphasizing mastering key technologies essential for data protection. Nevertheless, 20% of respondents express no specific data security concerns, and only 15% possess both critical technologies for data protection and practical implementation experience.

#### Infrastructure for AI research

Table 8 outlines AI research infrastructure state across research institutions. Nearly half (47%) are establishing AI infrastructure, while a similar proportion (46%) plan to but have not yet initiated. A small minority (7%) have successfully established and currently utilize AI systems, mainly focusing on structured image reports for lung cancer and other tumor-related diseases.

**Table 8:** Infrastructure for AI research in the research institutions group.

Characteristics	Percentage
Laboratory status of establishing structured imaging reports	
Preparing to establish	47%
Intend to initiate but have not yet started	46%
Already established and in active use	7%
Researchers’ awareness of AI research institutions or organization	
Unaware of establishment status	45%
No AI research institution or organization established	45%
Aware of existing AI research institutions and organizations	10%
Researchers’ awareness of departments for transforming results into practical applications	
Institution has relevant departments	45%
Lacks information about such departments	41%
No such department exists	14%

Regarding dedicated AI research departments or organizations establishment, there is notable lack of awareness and implementation among researchers. Forty-five percent are uncertain about such entities’ existence within their institutions. Similarly, 45% report no dedicated AI research department or organization exists, with only 10% confirming their presence. Concerning capability for transforming research outcomes into practical applications, opinions are divided. Forty-five percent acknowledge relevant departments’ presence within their institutions. However, the majority (55%) indicate lack of such departments, with 41% unsure of their existence and 14% explicitly stating their absence.

#### Current state of AI research and development

Table 9 provides insights into AI research and development landscape as perceived by respondents. A significant majority (64%) have familiarity with AI-related products through hearsay rather than direct usage. Meanwhile, 18% are actively engaged in ongoing AI research and development efforts. A smaller fraction (14%) has hands-on experience with AI products, and only 4% have contributed to research and development yielding tangible outcomes for the AI research community.

**Table 9:** Current state of AI research and development in the research institutions group.

Characteristics	Percentage
Experience with AI-Related Products	
Aware of AI-related products but have not utilized them	64%
Active involvement in ongoing research and development	18%
Have used related products	14%
Participated in research and development with relevant results	4%
Challenges in AI research process	
Difficulty transitioning into practical applications	53%
Substantial workload and manpower investment	52%
Lack of industry standards	4%
Credibility of AI products	18%
Unclear legal responsibilities between products and clinical practitioners	3%
Absence of relevant knowledge and perseverance	18%
Collaboration among healthcare systems, research institutions, and industry	37%
Data acquisition and processing	29%
Capital injection	14%
Algorithm support	12%
Policy support	7%

Research-to-product application transition presents notable hurdles. Over half (53%) highlight the substantial gap between research achievements and their conversion into practical applications as a primary challenge, underscoring difficulties in achieving rapid market readiness. Similarly, 52% point out intensive workload and significant human resource requirements needed for AI research and development. Other challenges include lack of industry standards (4%), questions surrounding AI product credibility (18%), unclear legal responsibilities between products and clinical practitioners (3%), and absence of relevant knowledge and perseverance (18%). Furthermore, 37% identify the main challenge as fostering effective collaboration among healthcare systems, research institutions, and industry. Data acquisition and processing are viewed as the most daunting challenge by 29%, overshadowing concerns related to capital investment (14%), algorithm support (12%), and policy facilitation (7%).

#### Future development in the research institutions group

Table 10 demonstrates development prospects of AI in healthcare from the research institutions group. A vast majority (86%) see significant value in further exploring AI products for healthcare, although 28% temper expectations with caution about short-term viability of practical applications. A minority (7%) view AI as a fad lacking practical utility, while 5% anticipate that AI will eventually supersede traditional imaging methods. Interest among researchers is notably high in lesion screening and detection (71%), disease diagnosis and prognosis analysis (66%), evaluating treatment effectiveness (58%), and medical education (36%).

**Table 10:** Future development in the research institutions group.

Characteristics	Percentage
Prospects of AI research	
Worthy of investigation	86%
Practical results not expected short term	28%
Viewed as a temporary trend	7%
Traditional methods may be replaced	5%
Areas of interest in AI	
Lesion screening and detection	71%
Disease diagnosis and prognosis analysis	66%
Treatment effectiveness	58%
Medical education	36%
Interest in collaboration	
With medical institutions	75%
With research institutions	71%
With enterprises	51%
With educational institutions	27%
Favor building own teams	17%
Resources expected from collaborators	
Published papers	65%
Research project design	40%
Algorithm research	30%
Product development	19%
Product usage training	19%
Resources expected from hospitals in collaborations	
Access to imaging and clinical data	85%
Image annotation	5%
Joint application for research projects	16.5%
Assistance with software transformation	13.4%
Knowledge of clinical project determination	17.4%
Research funding	16.4%
Expected main output of AI collaboration	
Research papers	67%
Patents	62%
AI Products	56%
Research funding	50%
Personal gains	26%
Expected time frame for research output	
Within 1 year	20%
Within 1–2 years	43%
Over 2 years	37%
Desired support from collaborators	
Collaboration platform with AI companies	69%
Introducing hospital projects and data for research	66%
Regular training-related workshops	64%

Collaborative endeavors are highly sought after, with 75% keen on partnering with healthcare systems and 71% advocating for increased collaboration between research institutions. Industry and educational institution collaborations attract support rates of 51 and 27%, respectively, with 17% preferring to develop their own teams. Regarding resources, researchers prioritize access to scientific publications (65%) and research grants (40%). Other valuable resources include algorithm research (30%), product development (19%), and product usage training (19%). For hospital collaborations, researchers express strong desire for access to imaging and clinical data (85%), image annotation (56%), support in applying for research projects (53%), software transformation assistance (47%), clinical project determination knowledge (46%), and research funding (39%).

Expected outcomes from AI collaborations are predominantly research papers (67%), followed by patents (62%), AI product developments (56%), research funding (50%), and personal achievements (26%). Regarding timeline, 80% anticipate research outputs will materialize over 1 year, with 43% expecting results within 1–2 years and 37% foreseeing outcomes beyond 2 years. A smaller group (20%) hopes for results within the first year. Most coveted support from collaborators includes establishing platforms for cooperation with AI companies (69%), integrating hospital projects and data into research (66%), and organizing regular workshops for knowledge exchange (64%).

## Discussion

This study provides comprehensive examination of current state and challenges of collaboration among healthcare systems, research institutions, and industry in AI research and development. Findings depict a landscape marked by substantial interest but hindered by notable barriers to effective collaboration and implementation.

Previous studies have documented AI’s technical capabilities in medical imaging, with diagnostic accuracy often exceeding 90% for specific tasks (Chen, 2025). However, research increasingly emphasizes that technical performance alone does not guarantee clinical adoption, with fewer than 10% of published AI algorithms progressing to clinical implementation (Lawrence et al., 2025). Literatures identified persistent collaboration challenges including misaligned incentives, communication barriers between technical and clinical teams, data accessibility issues, and regulatory uncertainties (Ogut, 2025; Ye et al., 2024). Recent systematic reviews of AI implementation, including work on AI-Based Software as a Medical Device (AI-SaMD), have highlighted universal barriers such as lack of standardization, data quality concerns, and clinical workflow integration difficulties (Ebad et al., 2025). Despite this growing body of literature, few large-scale empirical studies examine perspectives of multiple stakeholder groups simultaneously within a single healthcare system (Marrugo et al., 2025).

The impressive clinician response rate highlights strong interest in AI’s potential to transform healthcare. Yet, the stark contrast between this interest and actual engagement in AI-related research or development activities indicates systemic barriers. The fact that most hospitals lack structured imaging report systems—basic yet crucial infrastructure for AI integration—underscores a fundamental readiness gap for AI adoption across healthcare institutions. This gap is further evidenced by limited establishment of dedicated AI research departments, which are crucial for fostering innovation and translating research into clinical practice (Celi et al., 2022; Ye et al., 2023).

The demographic and professional profile of clinician respondents, predominantly those with bachelor’s degrees and substantial radiology experience, suggests a workforce theoretically well-positioned to contribute to AI advancements. However, minimal involvement in AI research or development activities, alongside scant production of tangible outcomes such as patents or internationally recognized research, indicates a disconnect between potential and actualized contribution. This disconnect may stem from reported lack of AI-specific knowledge and training, highlighting a critical intervention area (Paranjape et al., 2019).

Our findings resonate with challenges reported in other healthcare specialties and international contexts. Similar barriers to AI adoption, including lack of standardized protocols, data privacy concerns, and limited interdisciplinary collaboration have been documented in pathology (Bessen et al., 2025), dermatology (Koka and Burkhart, 2023), and ophthalmology (Taribagil et al., 2023). A systematic review of AI implementation identified comparable infrastructure gaps and training deficits among clinicians (Jha et al., 2025). However, the magnitude of these barriers appears particularly pronounced in our study, with 72% of hospitals lacking dedicated AI research departments compared to approximately 45% reported in North American surveys (Poon et al., 2025). International comparisons reveal both shared and context-specific challenges. While data privacy concerns are universal, the absence of industry standards appears more critical in China’s rapidly evolving regulatory environment compared to regions with established frameworks like the European Union’s Medical Device Regulation (Van Kolfschooten and Van Oirschot, 2024). Conversely, China’s centralized healthcare system may offer advantages for large-scale data sharing once appropriate frameworks are established, unlike the more fragmented systems in the United States (Ye, 2021a). The collaborative enthusiasm observed in our study (88% of clinicians seeking partnerships with research institutions) aligns with global trends toward ecosystem-based AI development, though actualization of these collaborations remains limited across all contexts studied.

Willingness among clinicians to share data for AI research, coupled with recognition of data anonymization and confidentiality importance, presents an opportunity for leveraging clinical insights for AI development. However, actual collaboration mechanisms and existing concerns around data privacy and security protocols suggest that more structured, transparent, and secure frameworks for data sharing are necessary to fully realize this potential (Stahl and Wright, 2018).

Our findings align with recent international research on AI healthcare implementation, including AI-SaMD (Ebad et al., 2025). Both our study and AI-SaMD literature identify absence of clear standards as a paramount barrier, with 65% of our clinicians citing lack of industry standards compared to regulatory uncertainty reported in 78% of AI-SaMD studies. Similarly, data-related challenges appear universal: 33% of our researchers report not acquiring valuable data from collaborations, paralleling data quality and availability concerns in 68% of AI-SaMD studies. The gap we document between high interest (90% of clinicians) and minimal actual engagement (74% not involved in AI research) extends beyond technical and regulatory barriers, highlighting the critical importance of capacity building, education, and collaborative framework development alongside regulatory standardization efforts (Ye, 2021b).

Findings regarding AI collaboration challenges, particularly absence of industry standards and legal guidelines, resonate with broader issues facing AI in healthcare. These challenges, along with reported gaps in relevant AI knowledge among clinicians (Ding et al., 2024), underscore the need for multifaceted approaches to address these barriers. Future healthcare AI developments, as anticipated by clinicians, emphasize lesion screening and detection, disease diagnosis, and prognosis analysis, pointing toward areas where collaboration between healthcare systems, research institutions, and industry could be most fruitful (Ye, 2020).

The research institutions perspective complements and expands understanding of the collaboration landscape (Amann et al., 2020). Focus on image classification, segmentation, and analysis among research institutions aligns with clinicians’ interests in diagnostic AI applications (Zhang W. et al., 2025). However, challenges in acquiring impactful data and limited collaboration with healthcare systems highlight systemic barriers to effective data exchange and utilization for AI development.

To navigate the intricate landscape outlined by our findings and effectively harness AI potential within healthcare, several strategic recommendations emerge as pivotal. First, addressing the critical gap in AI-specific knowledge among clinicians is paramount. This involves developing and disseminating targeted education and training programs that not only familiarize healthcare professionals with AI technologies but also equip them with skills to actively participate in AI research and development (Sapci and Sapci, 2020). Such initiatives could be spearheaded through collaborative efforts between educational institutions, healthcare systems, and industry partners, ensuring curriculum is both comprehensive and applicable to current clinical practices (Ye et al., 2020). Second, establishing robust, secure data-sharing frameworks is essential. These frameworks should prioritize patient privacy and data security while facilitating seamless information exchange between healthcare systems and research entities. By implementing standardized protocols and leveraging advanced encryption technologies, these frameworks can alleviate data privacy concerns and enhance collaboration efficiency (Gerke et al., 2020). Furthermore, these systems should be designed for interoperability, allowing integration of diverse data sources and thereby enriching data available for AI research (Panesar, 2019). Third, absence of clear industry standards and legal guidelines has been identified as a significant barrier to AI integration in healthcare. Thus, there is pressing need for regulatory bodies, in collaboration with healthcare professionals, researchers, and industry stakeholders, to develop comprehensive standards and guidelines (Shelmerdine et al., 2021). These regulations should address ethical considerations, data usage, and AI technology deployment, ensuring AI applications in healthcare are both safe and effective (Vasey et al., 2022; Ye, 2021c). Moreover, establishing legal frameworks can help clarify all parties’ responsibilities and protect patient interests, thereby fostering a more trustworthy environment for AI development (Chen and Ye, 2025).

Establishing dedicated AI research departments within healthcare institutions represents another vital step toward bridging the gap between potential and actualized AI advancements (Ye et al., 2025). These departments could serve as innovation hubs, facilitating AI research translation into clinical applications (Dwivedi et al., 2021). By fostering closer collaboration between clinicians and AI researchers, these departments can ensure AI developments align with clinical needs and are rapidly integrated into healthcare practices (Ye et al., 2022). Fostering multidisciplinary collaborations stands out as a vital strategy for advancing AI in healthcare (Gama et al., 2022). By bringing together expertise of healthcare professionals, AI researchers, and industry innovators, these collaborations can drive development of AI applications that are not only technologically advanced but also deeply attuned to clinical care complexities (Dwivedi et al., 2022; Ye and Sanchez-Pinto, 2020). Such partnerships should aim to leverage unique strengths and perspectives of each sector, ensuring AI technologies are developed in ways that are both innovative and grounded in real-world healthcare needs (Greenwood et al., 2021).

## Limitation

This study has several limitations. First, reliance on survey data, although providing valuable insights from a wide range of respondents, carries inherent limitations related to self-reporting, including potential response biases and accuracy of self-assessed knowledge and experiences. Despite efforts to ensure comprehensive and diverse respondent pools, findings may not fully capture the breadth of perspectives across all healthcare settings and geographical regions. Second, the study’s focus on China, while offering in-depth insights into collaborative landscape within a major healthcare and technological market, might limit findings’ generalizability to other contexts. Different countries may have unique regulatory environments, technological infrastructures, and cultural attitudes toward AI in healthcare, which could influence collaborative efforts’ nature and success in ways not captured by this study. Finally, the study’s quantitative approach, while effective for identifying broad trends and patterns, may not fully capture collaborative relationship complexities, interdisciplinary communication nuances, or qualitative aspects of innovation and problem-solving in AI research and development. Future research could benefit from incorporating qualitative methods such as interviews or case studies to gain deeper insights into these aspects.

## Conclusion

This study illuminated the complex and dynamic landscape of collaboration among healthcare systems, research institutions, and industry stakeholders in the AI domain. The enthusiastic response from clinicians underscores widespread recognition of AI’s potential to enhance patient care, improve diagnostic accuracy, and streamline healthcare operations. However, this enthusiasm is tempered by implementation challenges, including lack of structured imaging report systems, insufficient collaboration between key stakeholders, and dearth of dedicated AI research and development departments within hospitals. These challenges are further compounded by concerns over data privacy and security and absence of clear industry standards and legal guidelines, which collectively hinder seamless AI technology integration into clinical practice. The recommendations emanating from this study underscore the critical need for concerted efforts by all stakeholders to address identified challenges. By fostering collaborative ecosystems that encourage sharing of knowledge, resources, and expertise, we can accelerate the pace of AI innovation and its healthcare applications. Moreover, establishing comprehensive industry standards and legal frameworks will provide necessary foundations for building trust and credibility in AI technologies, ensuring their ethical and effective use in patient care.

## Data Availability

The original contributions presented in the study are included in the article/supplementary material, further inquiries can be directed to the corresponding author.

## References

[ref1] Albahri A. S. Duhaim A. M. Fadhel M. A. Alnoor A. Baqer N. S. Alzubaidi L. . (2023). A systematic review of trustworthy and explainable artificial intelligence in healthcare: assessment of quality, bias risk, and data fusion. Inf. Fusion 96, 156–191. doi: 10.1016/j.inffus.2023.03.008

[ref2] Allioui H. Mourdi Y. (2023). Unleashing the potential of AI: investigating cutting-edge technologies that are transforming businesses. Int. J. Comput. Eng. Data Sci. 3, 1–12. Available online at: https://www.ijceds.com/ijceds/article/view/59

[ref3] Amann J. Blasimme A. Vayena E. Frey D. Madai VI.. (2020). Explainability for artificial intelligence in healthcare: a multidisciplinary perspective. BMC Med. Inform. Decis. Mak. 20, 1–9. doi: 10.1186/s12911-020-01332-6 33256715 PMC7706019

[ref4] Bajwa J. Munir U. Nori A. Williams B. (2021). Artificial intelligence in healthcare: transforming the practice of medicine. Future Healthc. J. 8:e188. doi: 10.7861/fhj.2021-0095, 34286183 PMC8285156

[ref5] Bessen J. L. Alexander M. Foroughi O. Brathwaite R. Baser E. Lee L. C. . (2025). Perspectives on reducing barriers to the adoption of digital and computational pathology technology by clinical labs. Diagnostics 15:794. doi: 10.3390/diagnostics15070794, 40218144 PMC11988507

[ref6] Celi L. A. Cellini J. Charpignon M. L. Dee E. C. Dernoncourt F. Eber R. . (2022). Sources of bias in artificial intelligence that perpetuate healthcare disparities—a global review. PLOS Digit. Health 1:e0000022. doi: 10.1371/journal.pdig.0000022, 36812532 PMC9931338

[ref7] Chen Y. (2025). Artificial intelligence in diagnostic imaging: a review of accuracy and ethical challenges. Anfo Publication House.

[ref8] Chen H. Ye J. (2025). Digital health technology burden and frustration among patients with multimorbidity. medRxiv:2025.10.09.25337645. doi: 10.1101/2025.10.09.25337645 PMC13630286 42334194

[ref9] Chen H. Simmons W. Hashish M. A. Ye J. (2024). Telehealth utilization and patient experiences: the role of social determinants of health among individuals with hypertension and diabetes. medRxiv:2024.08.01.24311392. doi: 10.1101/2024.08.01.24311392 PMC13400121 42489378

[ref27] Chinese Society of Radiology. Available online at: https://csr.cma.org.cn/cn/index.aspx (Accessed June 30, 2019).

[ref10] Ding S. Ye J. Hu X. Zou N. (2024). Distilling the knowledge from large-language model for health event prediction. Sci. Rep. 14:30675. doi: 10.1038/s41598-024-75331-2, 39730390 PMC11680798

[ref11] Dwivedi Y. K. Hughes L. Baabdullah A. M. Ribeiro-Navarrete S. Giannakis M. al-Debei M. M. . (2022). Metaverse beyond the hype: multidisciplinary perspectives on emerging challenges, opportunities, and agenda for research, practice and policy. Int. J. Inf. Manag. 66:102542. doi: 10.1016/j.ijinfomgt.2022.102542

[ref12] Dwivedi Y. K. Hughes L. Ismagilova E. Aarts G. Coombs C. Crick T. . (2021). Artificial intelligence (AI): multidisciplinary perspectives on emerging challenges, opportunities, and agenda for research, practice and policy. Int. J. Inf. Manag. 57:101994. doi: 10.1016/j.ijinfomgt.2019.08.002

[ref13] Ebad S. A. Alhashmi A. Amara M. Miled A. B. Saqib M. (2025). Artificial intelligence-based software as a medical device (AI-SaMD): a systematic review. Healthcare 13:817. doi: 10.3390/healthcare13070817, 40218113 PMC11988595

[ref14] Gama F. Tyskbo D. Nygren J. Barlow J. Reed J. Svedberg P. (2022). Implementation frameworks for artificial intelligence translation into health care practice: scoping review. J. Med. Internet Res. 24:e32215. doi: 10.2196/32215, 35084349 PMC8832266

[ref15] Gerke S. Minssen T. Cohen G. (2020). “Ethical and legal challenges of artificial intelligence-driven healthcare” in Artificial intelligence in healthcare (London, United Kingdom: Elsevier), 295–336.

[ref16] Greenwood D. A. Litchman M. L. Isaacs D. Blanchette J. E. Dickinson J. K. Hughes A. . (2021). A new taxonomy for technology-enabled diabetes self-management interventions: results of an umbrella review. J. Diab. Sci. Technol. 16, 812–824. doi: 10.1177/19322968211036430, 34378424 PMC9264439

[ref17] Jha D. Durak G. Das A. Sanjotra J. Susladkar O. Sarkar S. . (2025). Ethical framework for responsible foundational models in medical imaging. Front Med (Lausanne), 12:1544501. doi: 10.3389/fmed.2025.1544501 40458639 PMC12128638

[ref18] Jiang F. Jiang Y. Zhi H. Dong Y. Li H. Ma S. . (2017). Artificial intelligence in healthcare: past, present and future. Stroke Vasc. Neurol. 2, 230–243. doi: 10.1136/svn-2017-000101, 29507784 PMC5829945

[ref19] Koka S. S.-A. Burkhart C. G. (2023). Artificial intelligence in dermatology: current uses, shortfalls, and potential opportunities for further implementation in diagnostics and care. Open Dermatol. J. 17:e187437222304140. doi: 10.2174/18743722-v17-e230505-2022-27

[ref20] Lawrence R. Dodsworth E. Massou E. Sherlaw-Johnson C. Ramsay A. I. G. Walton H. . (2025). Artificial intelligence for diagnostics in radiology practice: a rapid systematic scoping review. EClinicalMedicine 83:103228. doi: 10.1016/j.eclinm.2025.103228, 40474995 PMC12140059

[ref21] Lin-Greenberg E. (2020). Allies and artificial intelligence: obstacles to operations and decision-making (spring 2020). Texas Natl. Secur. Rev. doi: 10.26153/tsw/8866

[ref22] Marrugo J. J. V. Piñeros J. M. N. Rincon E. H. H. (2025). Evidence on the utility of artificial intelligence in the interpretation of diagnostic radiological images in low and middle-income countries: a scoping review. Acad. Radiol. doi: 10.1016/j.acra.2025.11.012 41298174

[ref23] Ogut E. (2025). Artificial intelligence in clinical medicine: challenges across diagnostic imaging, clinical decision support, surgery, pathology, and drug discovery. Clin. Pract. 15:169. doi: 10.3390/clinpract15090169, 41002784 PMC12468291

[ref24] Panesar A. (2019). Machine learning and AI for healthcare. Coventry, UK: Apress. Springer.

[ref25] Paranjape K. Schinkel M. Nannan Panday R. Car J. Nanayakkara P. (2019). Introducing artificial intelligence training in medical education. JMIR Med. Educ. 5:e16048. doi: 10.2196/16048, 31793895 PMC6918207

[ref26] Poon E. G. Lemak C. H. Rojas J. C. Guptill J. Classen D. (2025). Adoption of artificial intelligence in healthcare: survey of health system priorities, successes, and challenges. J. Am. Med. Inform. Assoc. 32, 1093–1100. doi: 10.1093/jamia/ocaf065, 40323320 PMC12202002

[ref28] Sapci A. H. Sapci H. A. (2020). Artificial intelligence education and tools for medical and health informatics students: systematic review. JMIR Med. Educ. 6:e19285. doi: 10.2196/19285, 32602844 PMC7367541

[ref29] Shelmerdine S. C. Arthurs O. J. Denniston A. Sebire N. J. (2021). Review of study reporting guidelines for clinical studies using artificial intelligence in healthcare. BMJ Health Care Informat. 28:e100385. doi: 10.1136/bmjhci-2021-100385, 34426417 PMC8383863

[ref30] Stahl B. C. Wright D. (2018). Ethics and privacy in AI and big data: implementing responsible research and innovation. IEEE Secur. Privacy 16, 26–33. doi: 10.1109/MSP.2018.2701164

[ref31] Taribagil P. Hogg H. D. J. Balaskas K. Keane P. A. (2023). Integrating artificial intelligence into an ophthalmologist’s workflow: obstacles and opportunities. Exp. Rev. Ophthalmol. 18, 45–56. doi: 10.1080/17469899.2023.2175672

[ref32] Van Kolfschooten H. Van Oirschot J. (2024). The EU artificial intelligence act (2024): implications for healthcare. Health Policy 149:105152. doi: 10.1016/j.healthpol.2024.105152, 39244818

[ref33] Vasey B. Nagendran M. Campbell B. Clifton D. A. Collins G. S. Denaxas S. . (2022). Reporting guideline for the early-stage clinical evaluation of decision support systems driven by artificial intelligence: DECIDE-AI. Nat. Med. 28, 924–933. doi: 10.1038/s41591-022-01772-9, 35585198

[ref34] Wang X. Ren Z. Ye J. (2025). Predicting survival time for critically ill patients with heart failure using conformalized survival analysis. AMIA Summits Transl. Sci. Proc. 2025:576. 40502254 PMC12150701

[ref35] Xiao Y. Liu S. (2019). Collaborations of industry, academia, research and application improve the healthy development of medical imaging artificial intelligence industry in China. Chin. Med. Sci. J. 34, 84–88. doi: 10.24920/003619, 31315748

[ref36] Ye J. (2020). The role of health technology and informatics in a global public health emergency: practices and implications from the COVID-19 pandemic. JMIR Med. Inform. 8:e19866. doi: 10.2196/19866, 32568725 PMC7388036

[ref37] Ye J. (2021a). Health information system's responses to COVID-19 pandemic in China: a national cross-sectional study. Appl. Clin. Inform. 12, 399–406. doi: 10.1055/s-0041-1728770, 34010976 PMC8133837

[ref38] Ye J. (2021b). “Design and development of an informatics-driven implementation research framework for primary care studies” in AMIA annual symposium proceedings (American Medical Informatics Association). PMC8861697 35308925

[ref39] Ye J. (2021c). The impact of electronic health record–integrated patient-generated health data on clinician burnout. J. Am. Med. Inform. Assoc. 28, 1051–1056. doi: 10.1093/jamia/ocab017, 33822095 PMC8068436

[ref40] Ye J. He L. Beestrum M. (2023). Implications for implementation and adoption of telehealth in developing countries: a systematic review of China’s practices and experiences. npj Digit. Med. 6:174. doi: 10.1038/s41746-023-00908-6, 37723237 PMC10507083

[ref41] Ye J. Sanchez-Pinto L. N. (2020). “Three data-driven phenotypes of multiple organ dysfunction syndrome preserved from early childhood to middle adulthood” in AMIA annual symposium proceedings (American Medical Informatics Association). PMC8075454 33936511

[ref42] Ye J. Woods D. Bannon J. Bilaver L. Kricke G. McHugh M. . (2022). Identifying contextual factors and strategies for practice facilitation in primary care quality improvement using an informatics-driven model: framework development and mixed methods case study. JMIR Hum. Factors 9:e32174. doi: 10.2196/32174, 35749211 PMC9269526

[ref43] Ye J. Zhang R. Bannon J. E. Wang A. A. Walunas T. L. Kho A. N. . (2020). Identifying practice facilitation delays and barriers in primary care quality improvement. J. Am. Board Family Med. 33, 655–664. doi: 10.3122/jabfm.2020.05.200058, 32989060

[ref44] Ye J. . (2024). The role of artificial intelligence in the application of the integrated electronic health records and patient-generated health data. medRxiv:2024.05.01.24306690. PMC11141850 38827061

[ref45] Ye J Bronstein S. Hai J. Hashish M. A. (2025). DeepSeek in healthcare: a survey of capabilities, risks, and clinical applications of open-source large language models. arXiv preprint arXiv:2506.01257. doi: 10.48550/arXiv.2506.01257

[ref46] Zhang Z. Wu Q. Ding S. Wang X. Ye J. (2025). Echo-vision-FM: a pre-training and fine-tuning framework for echocardiogram video vision foundation model. Nat Commun:2024.10.09.24315195. doi: 10.1038/s41467-025-66340-4 41381490 PMC12764486

[ref47] Zhang S. Ding S. Xu Z. Ye J. (2025). Machine learning-based mortality prediction in critically ill patients with hypertension: comparative analysis, fairness, and interpretability. medRxiv:2025.04.05.25325307. doi: 10.3389/frai.2025.1686378 PMC12738824 41459572

[ref48] Zhang W Luo L. He M. Hai J. Ye J. (2025). DescriptorMedSAM: Language-image fusion with multi-aspect text guidance for medical image segmentation. arXiv preprint arXiv, 2503, 13806. doi: 10.48550/arXiv.2503.13806 PMC12852884 41526554

